# Retinal Venule Coverage by Pericytes Decreases in Multiparous Mice in a Time-Dependent Manner Post-Delivery

**DOI:** 10.3390/ijms24043967

**Published:** 2023-02-16

**Authors:** Junie P. Warrington, Maria Jones-Muhammad, Rachael O. Thompson, Tyranny Pryor, Qingmei Shao, Manasa Gunturu

**Affiliations:** 1 Department of Neurology, University of Mississippi Medical Center, Jackson, MS 39216, USA; 2 Program in Neuroscience, University of Mississippi Medical Center, Jackson, MS 39216, USA

**Keywords:** parity, retinal vasculature, pericytes, sex, retired breeders

## Abstract

Structural changes in the retinal vasculature have been linked to increased cardiovascular risks and also change as a function of age. Because multiparity has been associated with poorer cardiovascular health scores, we hypothesized that changes in retinal vascular caliber would be observed in multiparous, compared to nulliparous, females and retired breeder males. Age-matched nulliparous (*n* = 6) and multiparous (*n* = 11, retired breeder females with 4 ± 1 litters), and male breeder (*n* = 7) SMA-GFP reporter mice were included for assessment of retinal vascular structure. Multiparous females had higher body mass, heart weight, and kidney weight compared to nulliparous mice, with lower kidney and higher brain weight compared to male breeders. There was no difference in number of retinal arterioles or venules, or arteriole or venule diameter among groups; however, venous pericyte density (number per venule area) decreased in multiparous vs. nulliparous mice and was negatively associated with the time since last litter and with age. Our results suggest that the time elapsed since delivery is an important factor to be considered in multiparity studies. Taken together, changes in vascular structure and potentially function, are time- and age-dependent. Ongoing and future work will determine whether structural changes are associated with functional consequences at the blood–retinal barrier.

## 1. Introduction

During pregnancy, the female body undergoes numerous physiological changes to adapt to increased blood volume and to nourish the growing placenta and fetal unit [1]. After delivery, further physiological changes occur to revert to pre-pregnancy physiology. Because of pregnancy-associated physiological adaptations, it has been hypothesized that multiparity (having more than one delivery) and repeated physiological adaptations can have protective or pathophysiological consequences later in life. Indeed, studies report decreased cardiovascular health scores with increasing parity [2]. Furthermore, a Korean study showed that women with five or more deliveries had decreased hippocampal volumes and had worse memory scores [3]. Interestingly, a preclinical mouse study showed protection from experimental stroke in multiparous versus nulliparous mice with smaller infarcts and reduced neuro-inflammation despite having features of increased metabolic risks [4]. Furthermore, the effects of multiparity are not limited to only females. Both multiparous men and women develop increased body mass index, increased odds of coronary heart disease [5], and increased risk of cardiovascular disease [6] with increased parity. Taken together, these studies point to increased risk of cardiovascular complications with multiparity, regardless of sex.

The retina has been described as a window into the brain [7] and is accessible to clinicians using non-invasive imaging techniques. Studies have shown that retinal vascular structure changes throughout pregnancy and is different in women with preeclampsia, a hypertensive pregnancy disorder, with changes persisting to at least 1 year postpartum [8,9,10]. While interesting, these studies did not assess the effect of parity or multiparity on retinal vascular structure, and whether there are sex differences in retinal vascular structure was not addressed. Moreover, beyond vascular caliber, studies did not assess changes in vascular-associated cells in the retina. The SMA-GFP OTO2-10 reporter mouse has been used to assess pericyte and vascular smooth muscle cell changes [11,12] in the brain following different insults. Pericytes are mural cells closely associated with the microvasculature [13,14] with important roles in endothelial proliferation [15], blood–brain barrier integrity [16], capillary contraction, and regulation of capillary blood flow [17]. Importantly, reduced pericyte coverage has been associated with various diseases, including diabetic retinopathy [18,19,20], preclinical experimental autoimmune encephalomyelitis (EAE) model of multiple sclerosis [21], and Alzheimer’s disease [22,23]. Thus, we assessed pericyte density on venules within the retina, as a potential indicator of vessel integrity. 

In this study, we characterized changes in retinal vascular structure in female mice who had undergone multiple pregnancies, their male retired breeder partners, and female mice who had never been pregnant. We also stratified the multiparous female data based on time since last delivery and assessed the retinal vascular endpoints. 

## 2. Results

### 2.1. General Characteristics of Mice

A summary of the general characteristics of the mice used in this study is included in Table 1. Overall, retired breeders (males and females were heavier and had heavier internal organs compared to the nulliparous female mice. Furthermore, male retired breeders had higher kidney weights and lower heart weights compared to multiparous females. 

### 2.2. Pregnancy History of Multiparous Females

Mice in the multiparous group had 4 ± 1 litters and were euthanized at 57 ± 35 days after the last delivery. Moreover, mice delivered 25 ± 10 pups with 15 ± 10 pups surviving to weaning, representing 57 ± 29% survival.

### 2.3. Retinal Vascular Structure

**Figure 1:**
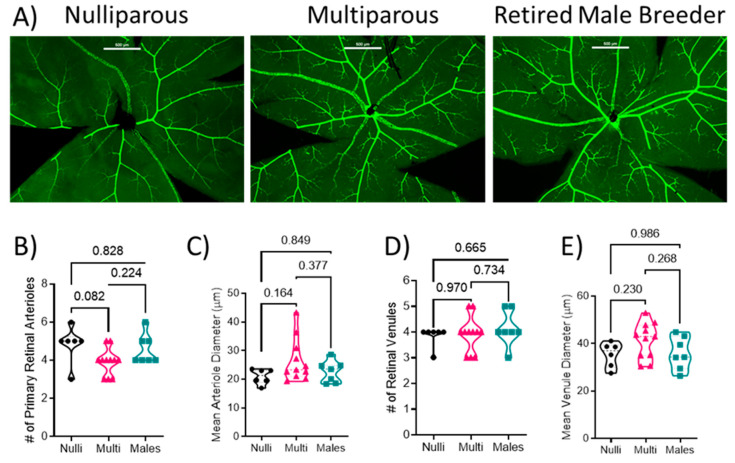
Quantification of retinal vascular parameters. (**A**) Representative images of the retinal mounts from each group. Scale bar represents 200 μm. (**B**) Number of primary arterioles, (**C**) arteriolar diameter, (**D**) number of primary venules, and (**E**) diameter of venules. Mean values from 1–2 retinal mounts per mouse are shown by individual points. Thick dashed line represents the median values. *p*-values are indicated for each post-hoc comparison. *n* = 6–11 per group. Mean arteriole diameter and number of venules were analyzed using Kruskal–Wallis test followed by Dunn’s multiple comparison test. All other data were analyzed using one-way ANOVA followed by Tukey’s post-hoc test.

Retinal flat mounts were imaged and the SMA-GFP+ blood vessels were analyzed (Figure 1A). There was no difference in the number of arterioles (F[2, 21] = 3.025; *p* = 0.070) between nulliparous (5 ± 1), multiparous (4 ± 1), or males (5 ± 1) (Figure 1B, *p* > 0.05) or arteriole diameter (F[2, 21] = 2.056; *p* = 0.153; 20.9 ± 2.4 µm in nulliparous, 26.4 ± 7.5 µm in multiparous, and 22.7 ± 3.8 µm in males) among the groups (Figure 1C). There was no difference in the number of venules among the groups (F[2, 21] = 0.438; *p* = 0.651; 4 ± 0 venules in nulliparous, 4 ± 1 in multiparous, and 4 ± 1 in males (Figure 1D). Moreover, there was no difference in venule diameter (F[2, 21] = 1.985; *p* = 0.162; 35.4 ± 4.8 in nulliparous, 41.3 ± 7.6 in multiparous, and 36.0 ± 6.9 µm in males) or arteriole diameter (F[2, 21] = 2.056; *p* = 0.153; 20.9 ± 2.4 µm in nulliparous, 26.4 ± 7.5 µm in multiparous, and 22.7 ± 3.8 µm in males) among the groups (Figure 1E).

### 2.4. Retinal Venous Pericyte Coverage

**Figure 2:**
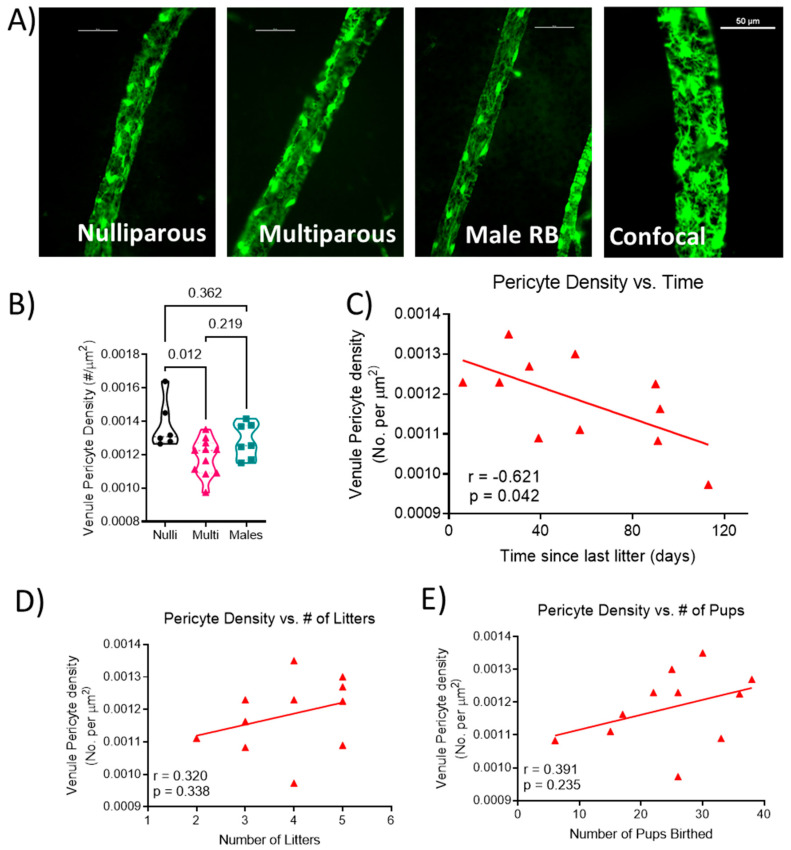
Quantification of venule coverage by pericytes. (**A**) Representative images from each group showing venule segments covered by pericytes. Unlabeled vessel segment represents a maximum projection image of z-stack obtained via confocal microscopy. Scale bar represents 50 μm. (**B**) pericyte density (number of pericytes per area of venule), relationship between (**C**) time since the last delivery, (**D**) number of litters, (**E**) number of pups birthed and venule pericyte density. Each data point represents the mean density of pericytes from a segment of each venule present in the retina per mouse. Thick dashed line represents the median. *n* = 6–11 mice per group. Pericyte density data were analyzed using one-way ANOVA followed by Tukey’s post-hoc test. Correlations were calculated using Pearson r correlation.

Representative images of venule segments from each group are shown in Figure 2A. Venule pericyte density was statistically different (F[2, 21] = 5.206; *p* = 0.015; Figure 2B), with lower density in multiparous females (0.0012 ± 0.0001) compared to nulliparous females (0.0014 ± 0.0001; *p* = 0.012) and not different from males (0.0013 ± 0.0001; *p* = 0.219). Correlation analysis showed a significant negative correlation (r = −0.621; *p* = 0.042; Figure 2C) between the length of time from the last delivery and venule pericyte density in the multiparous female mice. There was no significant relationship between the number of litters (r = 0.320, *p* = 0.338; Figure 2D) or the total number of pups delivered (r = 0.391, *p* = 0.235; Figure 2E) and venule pericyte density in multiparous females.

### 2.5. Impact of Length of Time Post-Delivery on General Characteristics

Because of the significant association between time since last delivery and retinal venule pericyte density, we separated multiparous mice into those with a post-delivery time of 35 days or less vs. those more than 35 days from the last delivery. Multiparous females that were more than 35 days from their last delivery had significantly lower heart weight and right kidney weights compared to those less than 35 days from their last delivery (Table 2).

### 2.6. Impact of Time since Last Delivery on Retinal Vascular Endpoints

**Figure 3:**
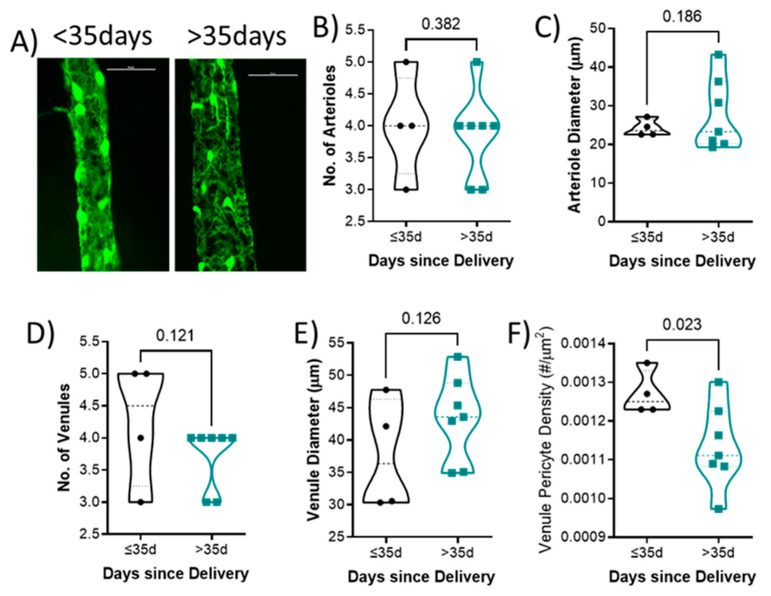
Quantification of retinal vascular parameters segregated by days since delivery. (**A**) Representative images of the retinal mounts from each group. Scale bar represents 50 μm. (**B**) Number of primary arterioles, (**C**) arteriolar diameter, (**D**) number of primary venules, and (**E**) diameter of venules, (**F**) venule pericyte density. Mean values from 1–2 retinal mounts per mouse are shown by individual points. Thick dashed line represents the median. *p*-values are indicated for each comparison. *n* = 6–11 per group. Data were analyzed using unpaired *t*-test.

Representative venules from each group is shown in Figure 3A. There was no difference in the number of arterioles (4 ± 1 vs. 4 ± 1; *p* = 0.382; Figure 3B), arteriole diameter (24.2 ± 2.2 vs. 27.7 ± 9.3 μm; *p* = 0.186; Figure 3C), number of venules (4 ± 1 vs. 4 ± 0; *p* = 0.121; Figure 3D), or venule diameter (37.7 ± 8.7 vs. 43.4 ± 6.6 μm; *p* = 0.126; Figure 3E) between groups. Mice who had delivered less than 35 days had higher retinal venule pericyte density (0.0013 ± 0.0001) compared to those with their last delivery more than 35 days (0.0011 ± 0.0001; *p* = 0.023; Figure 3F).

### 2.7. Relationship between Body Mass or Age on Retinal Vessel Parameters

**Figure 4:**
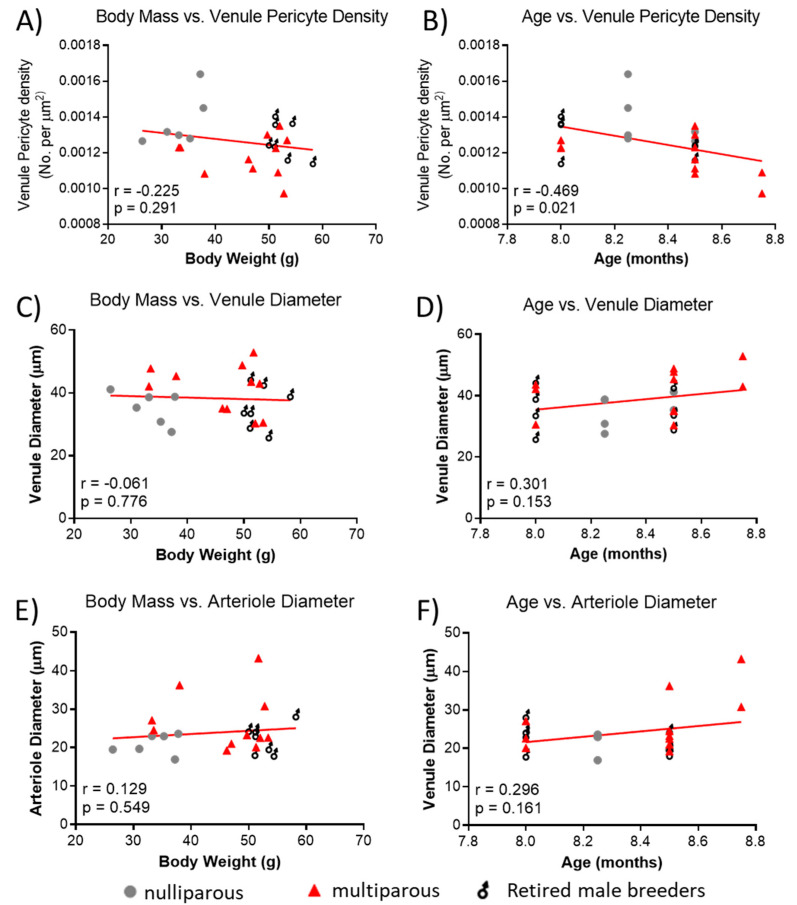
Relationship between body mass, age and retinal vascular parameters. (**A**) Association between body mass and retinal venule pericyte density. (**B**) Age vs. retinal venule pericyte density, (**C**) body mass vs. retinal arteriolar diameter, (**D**) age vs. venule diameter, (**E**) body mass vs. arteriole diameter, and (**F**) age vs. arteriole diameter. Mean values per mouse are shown by individual points. *n* = 6–11 per group. Lines were generated using linear regression. Pearson correlation was used for associations.

Because of the significant difference in body mass between groups and the reported association of age with retinal vascular parameters, we explored whether observed retinal vascular changes could be influenced by body mass and age. There was no significant relationship between body mass and venule pericyte density (Figure 4A), but age of mice was negatively associated with pericyte density (Figure 4B). There was no significant association between body mass (Figure 4C) or age (Figure 4D) and venule diameter. Lastly, there was no significant association between body mass (Figure 4E) or age (Figure 4F) and arteriole diameter.

## 3. Discussion

Retinal vascular structural changes have been reported to occur during normal pregnancy and the hypertensive pregnancy disorder, preeclampsia [1,8]. The current study is the first to report changes in retinal vascular structure in the context of multiparity. We report that while multiparity is associated with increased body and organ weights, there was no effect on the number of primary venules or arterioles and no influence on vascular diameter compared to nulliparous females or breeder male partners. Interestingly, the density of pericytes on retinal venules was significantly lower in the multiparous females compared to the nulliparous females, with a negative association between pericyte density and time since the last delivery/pregnancy. Moreover, when data from the multiparous group were segregated based on a cut-off last delivery of 35 days or less and more than 35 days, we found that the only retinal vascular parameter that was different was the venule pericyte density. A significant negative correlation between age and venule pericyte coverage was also discovered.

Few studies have assessed changes in retinal vascular structure during normal pregnancy. One such study used optical coherence tomography (OCT) on late pregnant women and non-pregnant controls and showed decreased superficial retinal capillary plexus perfusion density with no change in vessel length density [24]. In the current study, we found no difference in the number of primary arterioles or venules and no difference in diameter of these vessels with multiparity. Clinical studies have reported vasodilation during pregnancy and subsequent vasoconstriction in the postpartum period using OCT [25]. These studies focused on the retinal capillaries, while the current study focused on the larger venules and arterioles. In terms of the retinal venules and arterioles, Soma-Pillay and colleagues reported decreased diameter of both retinal arterioles and venules in patients with preeclampsia, which persisted in the postpartum period (up to 1 year, the length of the follow-up period) [8]. While not in the retina, other studies have looked at the effect of multiparity or repeated breeding on vascular function, and reported renal vasoconstriction and increased renal vascular resistance in old retired breeder female rats when compared to old virgin or young virgin female rats [26].

One of the key findings of our study was decreased coverage of venules by pericytes in the multiparous females and a negative correlation with the time elapsed since the last delivery. Pericytes have a role in strengthening the blood–brain/retinal-barrier [13,27,28,29,30], and other groups have proposed that the SMA-GFP reporter mouse, used in the current study, is a reliable model to study retinal pericytes [31]. Loss of retinal vascular pericytes is an early feature of diabetic retinopathy [19,20] as well as Alzheimer’s disease [22,23], and coincides with increased blood–retinal barrier permeability. Our findings hint to compromised blood–retina barrier in female mice after multiple pregnancies.

Another interesting finding is that retinal venule pericyte coverage decreased with age, independent of sex or parity, even though the age range of mice used in the study was small (less than 1-month difference between animals). This relationship was not observed for retinal arteriole and venule diameter. In humans, a 5-year increase in age was associated with decreased retinal venule and arteriole caliber, with women having higher arteriole diameters and no difference in venule caliber compared to men [32]. In our mice, we observed an increased venule and arteriole diameter with age, although it was not statistically significant. With a wider range of ages, and larger sample size, a significant association may have been observed. This will be explored in future studies.

Our finding of higher body weight and heavier organ weights in multiparous females and retired male breeders compared to nulliparous female is in line with epidemiological studies showing increased incidence of overweight with parity in a Mexico City cohort [33] and increased obesity with increased parity in a Chinese population [34]. We did not assess whether the excess body weight was being driven by increased fat in our study, but we hypothesize that the differences could be partly due to the difference in rodent chow available to the nulliparous females versus the designated long-term breeders. Interestingly, a British study showed that both men and women had increased body mass index with increased parity and that both sexes had an increased odds of coronary heart disease [5]. A similar finding was reported in an Iranian cohort of men and women, reporting increased risk of cardiovascular disease with increased parity [6]. Taken together, our findings of increased body weight in male and female retired breeders are consistent with epidemiological studies.

Our analyses revealed that retired male breeders had lower brain weight compared to nulliparous females. Our finding of lower brain weight in the males is in contrast with human data where male brains are reportedly larger than female brains [35]. The reason for this discrepant finding is unknown.

Our study has several strengths. We included a strong sample size of nulliparous, multiparous, and retired male breeder mice. We were able to conduct analyses within the multiparous group that involved the total number of litters, number of surviving offspring, and time since the last delivery, providing a robust analysis of pregnancy-related factors. Importantly, by using the SMA-GFP reporter mouse, we were able to easily differentiate between the arterioles and venules in the retina. Another strength of this study is that we were able to consider sex as a biological variable by including the male retired breeders in the analysis. Taken together, we have performed a systematic analysis of the retinal vascular structural changes associated with multiparity.

Our study has some limitations. First, we only assessed vascular parameters utilizing smooth muscle actin positive staining. Thus, we were not able to assess changes at the capillary level in any detail. Additionally, because the nulliparous and multiparous (retired breeder females and males) mice were fed different diets, some of the observed differences could have been driven by dietary differences. This is especially relevant for the differences in body weight. Additionally, because of the retrospective design of this study, we did not have access to age-matched virgin males for analysis of the retinal vasculature. We are, therefore, unable to fully address the effect of multiparity on the retinal vasculature in males. This will be addressed in future studies. Additionally, we did not have access to age-matched C57BL/6 nulliparous or multiparous mice for comparing to our SMA-GFP mice. It will be interesting to determine whether our observation is strain-dependent. Ongoing studies address changes in capillary structure in the retinal mounts and brains of nulliparous and multiparous mice. Future studies will assess whether there are changes in vascular function (perfusion and blood–brain/retinal barrier permeability) in the brains and retinas of mice and will correlate these to blood pressure.

## 4. Materials and Methods

### 4.1. Animals

To enable the direct visualization of blood vessels, a transgenic mouse model utilizing the *Acta2* promoter to direct the expression of green fluorescent protein (GFP) was used. These mice were generated by Dr. J.-Y. Tsai [31]. The design of the *Acta2* promoter was described by Wang et al. [36]. Homozygous pairs of smooth muscle α-actin (SMA)-GFP OTO2-10 mice, a generous gift from Dr. Robert Fariss (NEI, NIH) were used to establish a colony in the animal facilities at the University of Mississippi Medical Center (UMMC). Mice were maintained under normal 12 h light and 12 h dark conditions in a temperature- and humidity-controlled environment. All mice in this study were used for breeding purposes as long-term breeding pairs/trios (multiparous and males) or for short-term generation of timed pregnant mice for other studies (nulliparous) after unsuccessful mating (2–3 times). Breeder mice were maintained on a breeder diet (Teklad global 19% protein extruded rodent diet 2019) throughout the breeding period. The length of time from the last delivery of pups, total number of liters, number of pups birthed, and number of pups weaned were recorded for each female retired breeder. Because the male breeders were sometimes used in trio breeding with 2 females, we did not calculate these data for the males. Nulliparous females were fed normal rodent chow diet (Teklad 22/5 Rodent Diet 8640). All animal procedures were approved by the Institutional Animal Care and Use Committee at UMMC.

### 4.2. Study Design

This was a retrospective design utilizing collected tissues and breeding records kept in the lab for the transgenic reporter mouse line. Age-matched virgin males were not routinely kept in our animal facility as males are maintained specifically for breeding purposes by our group.

### 4.3. Tissue Collection

Under isoflurane anesthesia, mice were euthanized via collection of the heart in the morning. Organs (heart, kidneys, and brains) were removed and weighed. Eyes were carefully removed and stored in 4% paraformaldehyde overnight and transferred to 30% sucrose for at least 72 h until the eyes sunk to the bottom of the tube. Retinas were micro-dissected under a dissecting scope. Flat mounts were created and retinas were imaged immediately using a fluorescence microscope.

### 4.4. Retinal Mount Imaging and Analysis

**Figure 5:**
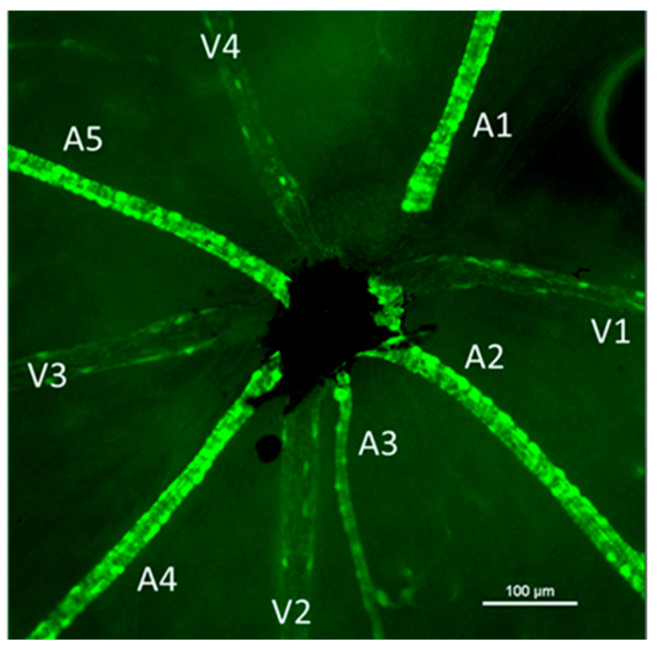
Representative image of the central retina showing the vascular branches identified by their smooth muscle actin staining. Each arteriole is labeled (A) and venules are identified with a (V). Arterioles had a brighter, more banded and continuous staining while venules had punctate cells on their surface. Images were captured using fluorescence microscopy. Scale bar represents 100 μm.

Images of the central retina were captured using a 4× objective lens for vessel counting (identified by GFP fluorescence). Higher magnification images were captured for each venule (identified based on the arrangement of the smooth muscle actin positive cells). Venules had punctate SMA-GFP+ cells along them while arterioles had brighter, banded, and continuous SMA-GFP staining (Figure 5). Using NIS Elements software, the diameter of each venule and arteriole was obtained at the area closest to the central retina (where the optic nerve leaves the eye). The number of SMA+ cells on the venule segments was counted and the area of the vessel segment was measured using the free-hand area tool in NIS Elements software. Density of pericytes was calculated as (number of pericytes/venule area) for one venule segment of each venule and averaged per retina.

### 4.5. Statistics

Normality of data was determined using the Shapiro–Wilk test. Normally distributed datasets were compared using one-way ANOVA and Tukey’s multiple comparison post hoc test while non-normally distributed data were compared using the Kruskal–Wallis test followed by Dunn’s multiple comparison post hoc test. Data are presented as mean ± standard deviation. Relationship between venule pericyte density and pregnancy history-related factors was calculated using 2-tailed Pearson r correlation. Lines were generated using linear regression model. All statistical differences were calculated using GraphPad Prism (version 9.4.1). Significance threshold was set to *p* < 0.05. All graphs were generated using GraphPad Prism.

## 5. Conclusions

In conclusion, we have shown that multiparity is associated with decreased coverage of venules by pericytes in the retina, a finding that is associated with time post-delivery of the last litter rather than with the total number of pregnancies (litters). Clinical and epidemiological studies should strongly consider the time since the last pregnancy as a critical factor when performing multiparity studies.

## Figures and Tables

**Table 1:** General characteristics of mice.

General Characteristics	Nulliparous (*n* = 6)	Multiparous (*n* = 11)	Breeder Males (*n* = 7)
Age (months)	8.3 ± 0.1	8.4 ± 0.3	8.2 ± 0.3
Body mass (g)	33.5 ± 4.3	46.3 ± 7.7 †	52.8 ± 2.8 †
Heart Weight (mg)	131 ± 21	189 ± 30 †	188 ± 29 †
Heart/Body Weight	3.9 ± 0.3	4.2 ± 1.3	3.6 ± 0.5
Right kidney (mg)	165 ± 15	222 ± 34 †	265 ± 27 †‡
Left kidney (mg)	156 ± 10	199 ± 27 †	256 ± 39 †‡
Kidney/Body Weight	9.7 ± 0.8	9.3 ± 2.1	9.9 ± 1.0
Brain Weight (mg)	470 ± 5	471 ± 18	450 ± 19 ‡
Brain/Body Weight	14.2 ± 2.0	10.5 ± 2.1 †	8.5 ± 0.3 †

† *p* < 0.05 vs. nulliparous. ‡ *p* < 0.05 vs. multiparous. Data analyzed using Kruskal–Wallis test and Dunn’s post hoc comparisons.

**Table 2:** General Characteristics of Mice used for data collection.

General Characteristics	≤35 Days (*n* = 4)	>35 Days (*n* = 7)	*p*-Value
Age (months)	8.3 ± 0.3	8.5 ± 0.3	0.082
Body Mass (g)	43.0 ± 11.2	48.0 ± 5.0	0.159
Heart Rate (bpm)	470 ± 14	486 ± 13	0.058
Heart Weight (mg)	210 ± 30	178 ± 26	**0.045**
Right Kidney (mg)	241 ± 9	211 ± 39	**0.046** ‡
Left Kidney (mg)	201 ± 29	198 ± 28	0.439
Brain Weight (mg)	469 ± 17	472 ± 20	0.384

‡ Data analyzed using unpaired one-tailed *t*-test with Welch’s correction. Bold: Indicate statistical significance.

## Data Availability

All raw data used in support of this manuscript are available from the corresponding author upon request.
